# Recipe Recommendation With Hierarchical Graph Attention Network

**DOI:** 10.3389/fdata.2021.778417

**Published:** 2022-01-12

**Authors:** Yijun Tian, Chuxu Zhang, Ronald Metoyer, Nitesh V. Chawla

**Affiliations:** ^1^Department of Computer Science and Engineering and Lucy Family Institute for Data and Society, University of Notre Dame, Notre Dame, IN, United States; ^2^Department of Computer Science, Brandeis University, Waltham, MA, United States

**Keywords:** recipe recommendation, user behavior modeling, recipe graph, food, graph attention network

## Abstract

Recipe recommendation systems play an important role in helping people find recipes that are of their interest and fit their eating habits. Unlike what has been developed for recommending recipes using content-based or collaborative filtering approaches, the relational information among users, recipes, and food items is less explored. In this paper, we leverage the relational information into recipe recommendation and propose a graph learning approach to solve it. In particular, we propose *HGAT*, a novel hierarchical graph attention network for recipe recommendation. The proposed model can capture user history behavior, recipe content, and relational information through several neural network modules, including type-specific transformation, node-level attention, and relation-level attention. We further introduce a ranking-based objective function to optimize the model. Thorough experiments demonstrate that *HGAT* outperforms numerous baseline methods.

## 1. Introduction

Large-scale food data offers rich knowledge about food and can help tackle many central issues of human society (Mouritsen et al., 2017; Min et al., 2019; Tian et al., 2021). Recipe websites, in particular, contain a large volume of food data because individuals are eager to share their created recipes online (Teng et al., 2012). This provides an opportunity for other users to rate and comment, which helps people form the habit of referring to these websites when deciding what to eat (Ueda et al., 2011). Food.com^1^, one of the largest recipe-sharing websites in the world, collects over half a million recipes. This large volume of data also reflects the great demand for recipe-providing services (Ueda et al., 2011). Accordingly, digging into this overwhelming amount of online recipe resources to find a satisfying recipe is always hard (Britto et al., 2020), especially when recipes are associated with various heterogeneous content such us ingredients, instructions, nutrients, and user feedback. However, Recipe Recommendation Systems have the power to help users navigate through tons of online recipe data and recommend recipes that align with users' preferences and history behavior (Khan et al., 2019).

Existing recipe recommendation approaches are mostly based on the similarity between recipes (Yang et al., 2017; Chen et al., 2020). A few of the approaches tried to take the user information into account (Freyne and Berkovsky, 2010; Forbes and Zhu, 2011; Ge et al., 2015; Vivek et al., 2018; Khan et al., 2019; Gao et al., 2020), but they only defined similar users based on the overlapping rated recipes between users, while ignoring the relational information between users, recipes, or ingredients. Nevertheless, user preference toward food is complex. A user may decide to try a new recipe because of its ingredients, its flavor, or a friend's recommendation. Therefore, a thoughtful recipe recommendation should take all these factors into account. Thus, it is important to encode the relational information and deeply understand the relationship among users, recipes, and ingredients for recipe recommendation.

**Figure 1:**
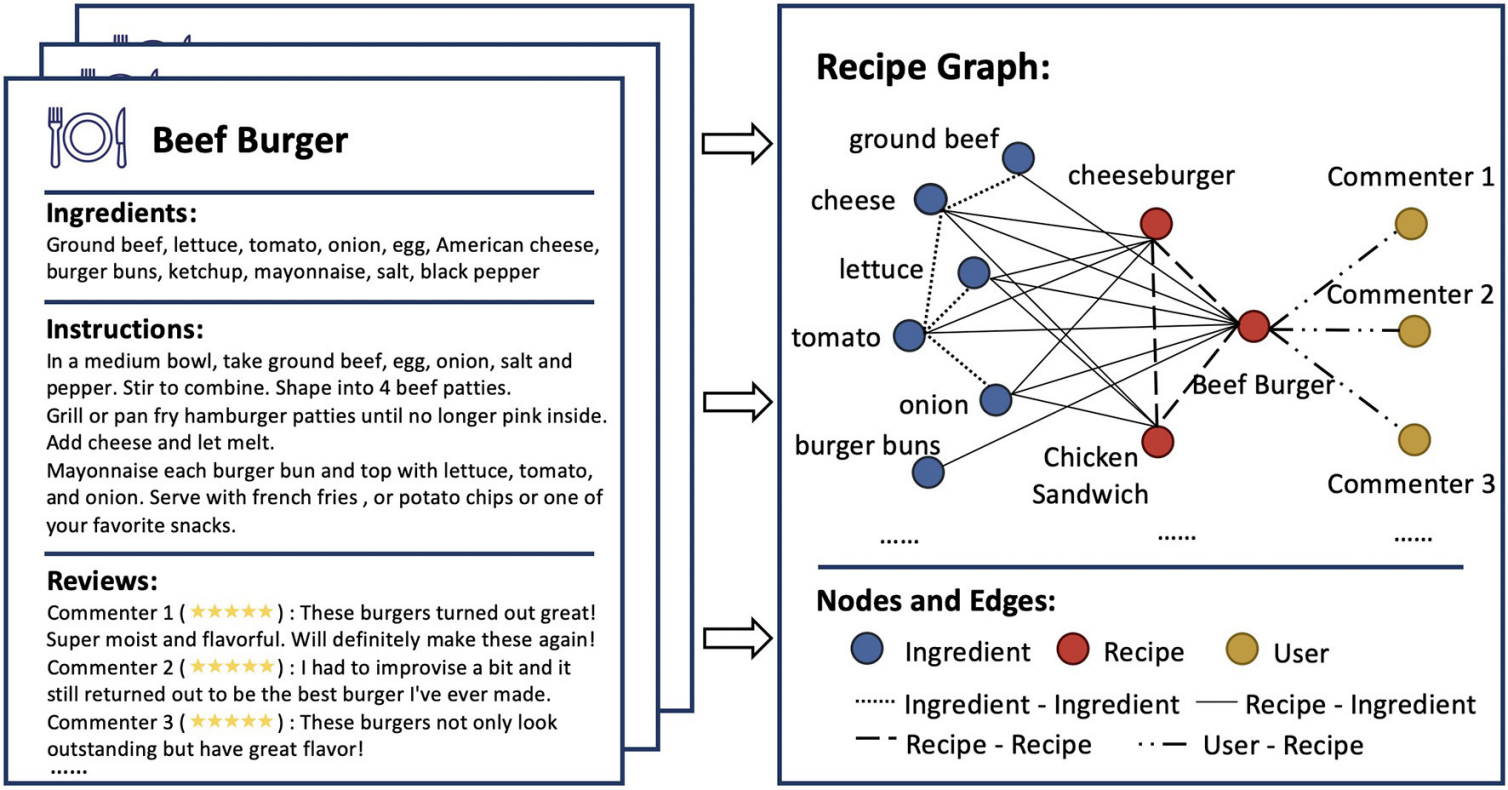
Illustration of Recipe Graph. Recipe examples with ingredients, instructions, and user reviews are shown on the **(left)**. The Recipe Graph **(right)** includes three types of nodes (i.e., ingredient, recipe, and user) and four types of relations which connect these nodes (i.e., ingredient-ingredient, recipe-ingredient, recipe-recipe, and user-recipe relations).

In this paper, we seek to leverage the relational information into recipe recommendation. We first construct a heterogeneous recipe graph to formulate the relationship among nodes. In particular, we start by collecting a corpus of recipes, where each recipe contains ingredients, instructions, and user ratings. We then transform this set of recipes into a recipe graph, with three types of nodes (i.e., ingredient, recipe, and user) and four types of relations connecting them (i.e., ingredient-ingredient, recipe-ingredient, recipe-recipe, and user-recipe relations). The illustration of the built recipe graph is shown in Figure 1. After constructing the recipe graph, we propose to solve the recipe recommendation problem using the graph learning approach, which naturally incorporates the relational information into recommendation.

In particular, we propose a novel heterogeneous recipe graph recommendation model, *HGAT*, which stands for Hierarchical Graph Attention Network. *HGAT* can recommend recipes to users that align with their history behavior and preferences. Specifically, we leverage several neural network modules to encode the recipe history, recipe content, and relational information. Specifically, we first apply a type-specific transformation matrix to model the heterogeneous content associated with each node (e.g., instructions and nutrients) and transform them into a shared embedding space. Then, we design a node-level attention module to encode the neighbor nodes with different weights. Considering we have multiple types of nodes and edges, the module individually runs for each relation to formulate relation-specific embeddings that contain each type of neighbor nodes information. For example, given a recipe node with three connected relations (i.e., recipe-ingredient, recipe-recipe, and recipe-user), the node-level attention module encodes each type of node individually and learns three relation-specific embeddings. Next, we develop a relation-level attention module to combine all the generated relation-specific embeddings with different weights and obtain the updated embedding for each node. To illustrate with the same example, the relation-level attention module merge the learned three relation-specific embeddings into one to represent the final embedding of the given recipe node. Therefore, the learned embeddings contain not only the neighbor nodes' information but also the connected relation information. Finally, we introduce a score predictor and a ranking-based objective function based on the learned user and recipe embeddings to optimize the model. To summarize, our main contributions in this paper are as follows:

- We argue that relational information is important in understanding user preference toward recipes. We further leverage this information into the recipe recommendation problem and proposed a graph learning approach to solve it.
- We develop *HGAT*, a hierarchical graph attention network for recipe recommendation. *HGAT* is able to capture both node content and relational information and make appropriate recommendations. *HGAT* comprises several neural network modules, including type-specific transformation, node-level attention, and relation-level attention.
- We conduct extensive experiments to evaluate the performance of our model. The results show the superiority of *HGAT* by comparing with a number of baseline methods for recipe recommendation.

The rest of the paper is organized as follows. Section 2 reviews the related work. Section 3 describes the proposed model. Section 4 presents the experiments of different models on recipe recommendation, followed by the conclusion in section 5.

## 2. Related Work

This work is closely related to the studies of food recommendation, recipe recommendation, and graph representation learning.

**Food Recommendation**. Food recommendation aims to provide a list of food items for users that meet their preference and personalized needs, including restaurants, individual food items, meals, and recipes (Trattner and Elsweiler, 2017a; Min et al., 2020). Despite food recommendation being a comparatively understudied research problem, a decent body of literature exists (Trattner and Elsweiler, 2017a). For example, Sano et al. (2015) used the transaction data in a real grocery store to recommend grocery items. Trattner and Elsweiler (2017b) used nine prominent recommender algorithms from the LibRec^2^ framework to recommend meal plans and recipes. In our work, we mainly focus on recipe recommendation since this is the one that is most relevant to our daily life.

Existing recipe recommendation approaches are mostly content-based, namely, recommending recipes based on the similarity between recipes (Yang et al., 2017; Chen et al., 2020). A few of the approaches proposed include user information into the recommendation procedures (i.e., collaborative filtering). Still, they only considered similar users based on the overlapping rated recipes, ignoring the relational information among users, recipes, or ingredients (Freyne and Berkovsky, 2010; Forbes and Zhu, 2011; Ge et al., 2015; Vivek et al., 2018; Khan et al., 2019; Gao et al., 2020). For example, Yang et al. (2017) developed a framework to learn food preference based on the item-wise and pairwise recipe image comparisons. Ge et al. (2015) utilized a matrix factorization approach that fuses user ratings and tags for recipe recommendation. On the other side, several works tried to recommend recipes based on a built graph, but the user information is not included (Li et al., 2010; Teng et al., 2012; Adaji et al., 2018). For example, Adaji et al. (2018) recommended recipes to users based on a graph where 2 recipes are connected if the same person has reviewed them. Li et al. (2010) constructed a cooking graph where the nodes are cooking actions or ingredients and recommend recipes based on their similarity. Teng et al. (2012) build two types of ingredient graphs to predict recipe pairs based on the substitution or complement of ingredients. Haussmann et al. (2019) leveraged a knowledge base question answering approach to recommend recipes based on the ingredients. In our work, we try to model the relational information through a heterogeneous recipe graph with user information included. Therefore, the graph learning approach could automatically encode the relational information and make considerate recommendations accordingly.

**User Behavior Modeling**. User Behavior Modeling is widely studied in the domain of recommendation. For example, Zhou et al. (2018) proposed an attention based user behavior modeling framework that projects all types of behaviors into multiple latent semantic spaces for recommendation. Elkahky et al. (2015) used a rich feature set to represent users, including their web browsing history and search queries to propose a content-based recommendation system. Abel et al. (2011) analyzed how user profiles benefit from semantic enrichment and compared different user modeling strategies in a personalized news recommendation system. In the field of food recommendation, Zhang et al. (2016) adopted user feedback of dining behavior to recommend restaurants. Musto et al. (2020) developed a recommendation strategy based on knowledge about food and user health-related characteristics by focusing on personal factors such as the BMI of users and dietary constraints. Our work incorporates user history behavior and user feedback such as the ratings toward recipes, which make our recommendation accounts for user interest and preferences.

**Graph Representation Learning**. Graph representation learning has become one of the most popular data mining topics in the past few years (Wu et al., 2021). Many graph representation learning approaches (Perozzi et al., 2014; Dong et al., 2017; Hamilton et al., 2017; Kipf and Welling, 2017; Schlichtkrull et al., 2018; Velickovic et al., 2018) were proposed to encode the graph-structure data. They take advantage of the content information associated with each node and the relational information in graph to learn vectorized embeddings, which are used in various graph mining tasks such as recommendation. For example, DeepWalk (Perozzi et al., 2014) learned node embeddings by feeding a set of random walks into a SkipGram model (Mikolov et al., 2013). Nodes in the graph were trained on each walk simultaneously where the neighbor nodes served as the contextual information. matapath2vec (Dong et al., 2017) conducted meta-path based random walks and utilized a SkipGram model to embed the heterogeneous graph. GAT (Velickovic et al., 2018) utilized an attention mechanism into message passing to learn node embeddings on homogeneous graphs by aggregating neighbor nodes' features with different attentions. In our work, we propose a hierarchical graph attention network that leverages attention mechanisms on different levels (i.e., node-level and relation-level), which can be applied on heterogeneous graphs and achieve outstanding performance.

## 3. Proposed Model

**Figure 2:**
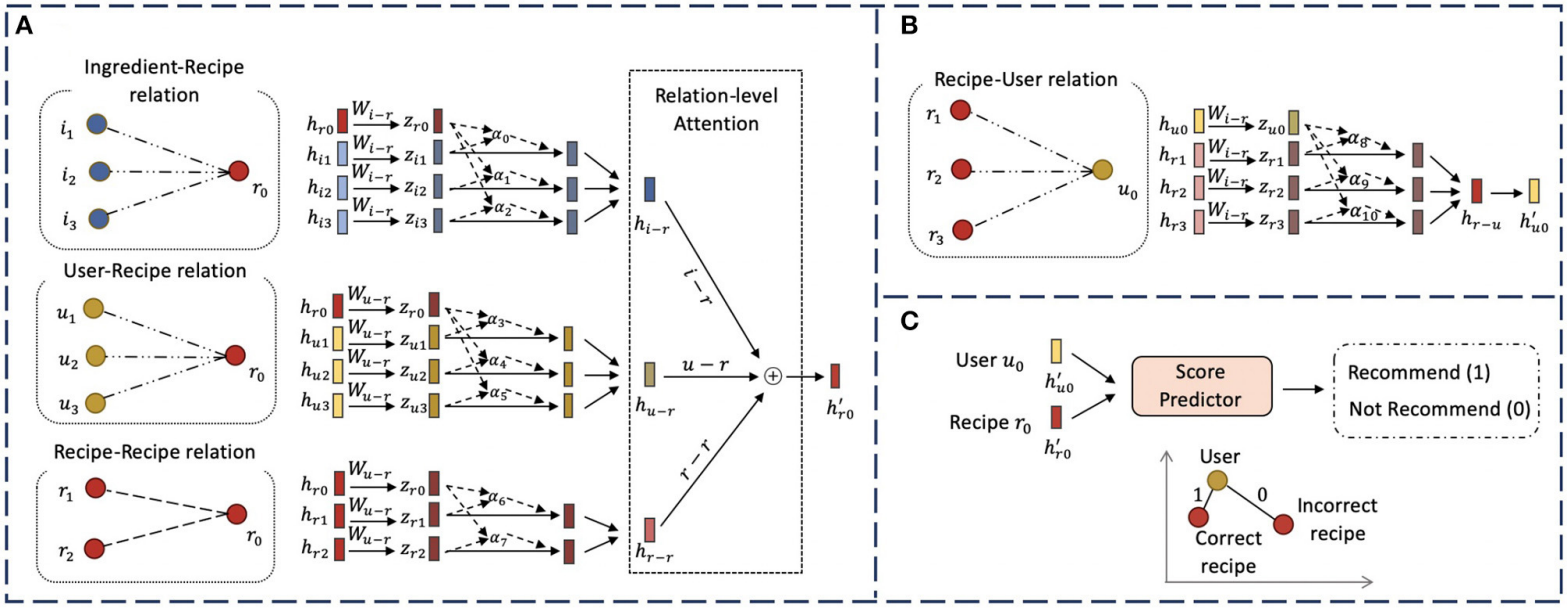
The overall framework of proposed *HGAT* model for recipe recommendation. **(A)** Illustration of node-level attention and relation-level attention for generating the embedding of recipe node *r*_0_; **(B)** Illustration of node-level attention for obtaining the embedding of user node *u*_0_. Relation-level attention is omitted here since user nodes only connect to other nodes through one relation; **(C)** The recommendation procedure based on the learned embeddings of user *u*_0_ and recipe *r*_0_.

In this section, we describe our novel hierarchical graph attention network model, *HGAT*. As illustrated in Figure 2, our model contains several major components. We first apply a type-specific transformation matrix to take various input features and project them into a shared embedding space. We then design a node-level attention module to encode the neighbor nodes that are connected by each relation to learn relation-specific embeddings. Next, we develop a relation-level attention module to combine all relation-specific embeddings and obtain the updated embedding for each node. Finally, we introduce a score predictor and a ranking-based objective function to optimize the model.

### 3.1. Type-Specific Transformation

Previous works (Salvador et al., 2017; Marin et al., 2019) have shown that cooking instructions are necessary for providing a discriminative understanding of the cooking process. We follow Marin et al.'s work (Marin et al., 2019) to use the pretrained skip-instruction embeddings as the instruction representations, and then average these representations to get one input raw feature for each recipe. For example, given a recipe with *M* sentence instruction embeddings {*x*_*ins*, 1_, *x*_*ins*, 2_, …, *x*_*ins, M*_}, we calculate the average instruction embeddings *x*_*ins*_ as the input feature for recipe nodes:


(1) $$\begin{array}{l} x_{ins}=\frac{1}{M}\left(\underset{i\in M}{\sum }x_{ins,i}\right), \end{array}$$

In addition, we formulate the nutrients of each ingredient into a vector and then use this nutrient vector as the content information for ingredient nodes. We denote this content information as the input feature *x*_*ing*_ for ingredient nodes. To represent the user nodes, since most of the users in *Food.com* have not provided any detailed information about themselves (e.g., description, location, preference, or demographic information) and it might violate the privacy policy by crawling this individual information, we use the xavier normal random initialized feature (Glorot and Bengio, 2010) as the input feature for user nodes, denoted as *x*_*user*_.

Due to the heterogeneity of input features for different node types (i.e., recipe, ingredient, and user), given a node *v*_*i*_ with type ϕ_*i*_, we introduce a type-specific transformation matrix *W*_ϕ_*i*__ to project the input features into the same embedding space. Specifically, for each node *v*_*i*_, we have the projection process formulated as follows:


(2) $$\begin{array}{l} x_{i}=\{\begin{array}{ll} x_{ins}, & \text{if }\phi_{\text{i}}\text{= recipe}\\ x_{ing}, & \text{if }\phi_{\text{i}}\text{= ingredient}\\ x_{user}, & \text{if }\phi_{\text{i}}\text{= user} \end{array}\\ h_{i}=W_{\phi_{i}}\cdot x_{i}, \end{array}$$

where $W_{\phi_{i}}\in {\mathbb{R}}^{d_{\phi_{i}}\times d}$ is the transformation matrix, *x*_*i*_ is the input feature of *v*_*i*_ with dimension *d*_ϕ_*i*__, and *h*_*i*_ is the projected feature of *v*_*i*_ with dimension *d*. In other words, with this type-specific transformation operation, the instruction, ingredient, and user embeddings would be in shared embedding space, and the model can therefore take arbitrary types of input features.

### 3.2. Node-Level Attention

**Figure 3:**
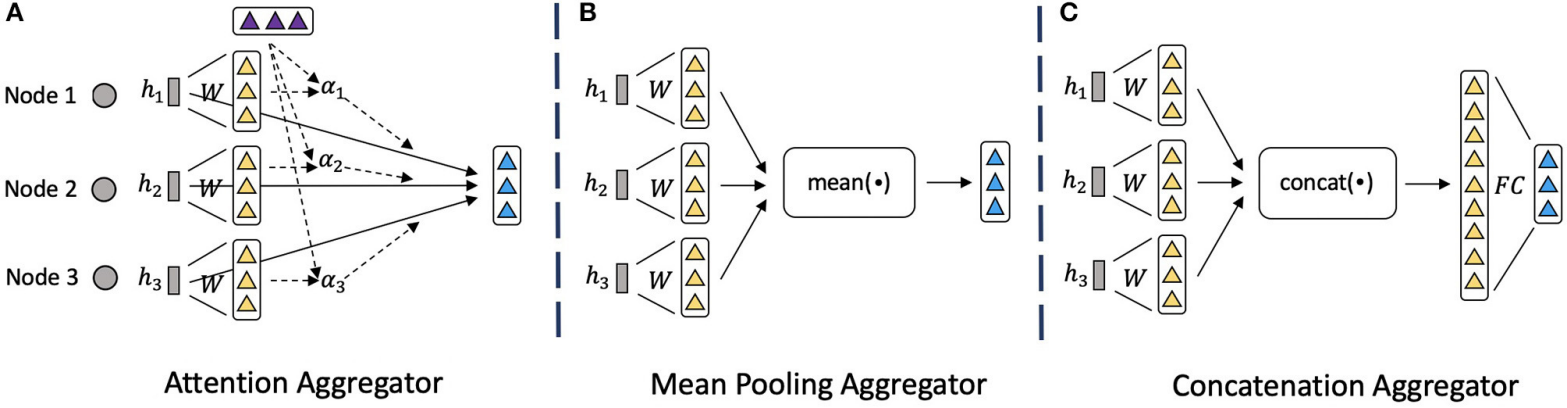
Illustrations of different neighbor aggregators. **(A)** Attention mechanism to encode neighbor nodes information. **(B)** Mean pooling operator to encode neighbor information. **(C)** Concatenation operator followed by linear transformation to encode neighbor information.

To encode and fuse the neighbor nodes information, we propose the node-level attention module, which is based on the attention aggregator as shown in Figure 3A. Compared to the mean pooling aggregator (Figure 3B) and concatenation aggregator (Figure 3C) that have been used widely (Hamilton et al., 2017) but simply combine the features, attention aggregator can learn the importance of each neighbor node and fuse them wisely. Specifically, for a node *v*_*i*_, we first use the node-level attention to calculate each relation-specific embedding *h*_*i, r*_ for each relation *r* that connects to *v*_*i*_. To explain how we get the relation-specific embeddings *h*_*i, r*_, we will start by describing a single node-level attention layer, as to calculate the node-level attention for each node. To compute the *h*_*i, r*_ in layer *l*+1, the input of the node-level attention layer is a set of node embeddings from layer *l*: $\left\{h_{1},h_{2},\ldots ,h_{N_{i,r}}\right\}\in {\mathbb{R}}^{d_{l}}$, where *N*_*i, r*_ denotes the number of neighbor nodes that connect to *v*_*i*_ through relation *r*, and *d*_*l*_ is the dimension of embeddings in layer *l*. In order to acquire sufficient expressive power to transform the input features into higher-level features $z_{i}\in {\mathbb{R}}^{d_{l+1}}$, where *d*_*l*+1_ is the dimension of embeddings in layer *l*+1, a shared linear transformation weight matrix $W_{r}\in {\mathbb{R}}^{d_{l}\times d_{l+1}}$ for relation *r* is applied:


(3) $$\begin{array}{l} z_{i}=W_{r}\cdot h_{i}. \end{array}$$

With the intermediary features *z*_*i*_, *z*_*j*_ for nodes *v*_*i*_ and *v*_*j*_, respectively, we calculate the unnormalized attention score *e*_*ij*_ between *v*_*i*_ and *v*_*j*_ to indicate the importance of *v*_*j*_ to *v*_*i*_. The calculation process is defined as follows:


(4) $$\begin{array}{l} e_{ij}=\text{LeakyReLU}\left[W_{ij}\cdot \left(z_{i}||z_{j}\right)\right], \end{array}$$

where || indicates the concatenation operator and $W_{ij}\in {\mathbb{R}}^{2d_{l+1}}$ is a weight vector that represents the attention between *v*_*i*_ and *v*_*j*_. Theoretically, our model can calculate the attention of every node to every other node without considering the relational information. Taking the message passing protocol into consideration, we acknowledge the graph structure and perform masked attention (Velickovic et al., 2018), which only computes *e*_*ij*_ if there exists an edge between *v*_*i*_ and *v*_*j*_ in the graph. In other words, we focus only on the first-order neighbor nodes of *v*_*i*_ (including *v*_*i*_). We further normalize the *e*_*ij*_ using the *softmax* function to make the coefficients easily comparable across different nodes. The normalized node-level attention vector α_*ij*_ is computed as:


(5) $$\begin{array}{l} \alpha_{ij}=\frac{\exp\left(e_{ij}\right)}{\underset{k\in N_{i,r}}{\sum }\exp\left(e_{ik}\right)}, \end{array}$$

After that, we use α_*ij*_ as coefficients to linearly combine the neighbor nodes features and generate the relation-specific embedding *h*_*i, r*_: The process is formulated as follows:


(6) $$\begin{array}{l} h_{i,r}=\sigma \left(\underset{j\in N_{i,r}}{\sum }\alpha_{ij}\cdot z_{j}\right), \end{array}$$

where σ is the nonlinear activation function (we use *ReLU* in our experiment). Instead of simply performing a single attention function, inspired by previous work (Vaswani et al., 2017), we extend the node-level attention to multi-head attention so that the model and the training process are more stable. In particular, we compute the node-level attention *M* times in parallel, and then concatenate the output and project them into a final learned relation-specific embedding *h*_*i, r*_. The computation process is formulated as follows:


(7) $$\begin{array}{l} h_{i,r}=\overset{M}{\underset{m=1}{∥}}\sigma \left(\underset{j\in N_{i,r}}{\sum }\alpha_{ij}\cdot z_{j}\right)W_{O}, \end{array}$$

where $W_{O}\in {\mathbb{R}}^{Kd_{m}\times d_{l+1}}$ is a learnable weight matrix, and *d*_*m*_ is the dimension of attention heads with *d*_*m*_ = *d*_*l*+1_/*M*. Therefore, with the reduced dimension *d*_*k*_ of each head, the total cost of computation for multi-head attention is similar to that of single-head attention with dimension *d*_*l*+1_.

### 3.3. Relation-Level Attention

In our graph, nodes are connected to other nodes through multiple types of relations (e.g., recipe to user, recipe to ingredient, and recipe to recipe), while each relation-specific node embedding can only represent the node information from one perspective. Therefore, the relation-specific node embeddings around a node need to be fused to learn a more comprehensive node embedding. To address the challenge of selecting relation and fusing multiple relation-specific node embeddings, we propose a relation-level attention module to learn the importance of different relations and automatically fuse them. Specifically, we use the relation-level attention module to combine all relation-specific embeddings *h*_*i, r*_ and generate the final node embedding *h*_*i*_.

We first use a shared nonlinear weight matrix *W*_*R*_ to transform the relation-specific node embeddings. Then we use a relation-level intermediary vector *q* to calculate the similarity with transformed embeddings, which is also used as the importance of each relation-specific node embedding. After that, we average the importance of all relation-specific node embeddings for relation *r* to generate the importance score *w*_*i, r*_ for node *v*_*i*_. The process is shown as follows:


(8) $$\begin{array}{l} w_{i,r}=\frac{1}{\left|V_{r}\right|}\underset{i\in V_{r}}{\sum }q^{T}\cdot \tanh\left(W_{R}\cdot h_{i,r}+b\right), \end{array}$$

where $W_{R}\in {\mathbb{R}}^{d_{l+1}\times d_{l+1}}$ is a nonlinear weight matrix, $b\in {\mathbb{R}}^{d_{l+1}}$ is the bias vector, $q\in {\mathbb{R}}^{d_{l+1}}$ is the relation-level intermediary vector, and *V*_*r*_ denotes the set of nodes under relation *r*. To make the coefficients comparable across different relations, we normalize *w*_*i, r*_ to get the relation-level attention vector β_*i, r*_ for each relation *r* using the *softmax* function. The normalization process is formulated as:


(9) $$\begin{array}{l} \beta_{i,r}=\frac{\exp\left(w_{i,r}\right)}{\underset{r\in R_{i}}{\sum }\exp\left(w_{i,r}\right)}, \end{array}$$

where *R*_*i*_ indicates the associated relations of node *v*_*i*_. Here, the generated attention vector β_*i, r*_ can be explained as the contribution of relation *r* to node *v*_*i*_. Apparently, the higher the β_*i, r*_, the more important the relation *r* is. Since different relations may contribute differently to the training objective, the relation-level attention vector for each relation could have different weights accordingly. Therefore, we fuse the relation-specific node embeddings *h*_*i, r*_ with the relation-level attention to obtain the final node embedding *h*_*i*_. The process is demonstrated as follows:


(10) $$\begin{array}{l} h_{i}=\overset{R_{i}}{\underset{r=1}{\sum }}\beta_{i,r}\cdot h_{i,r}. \end{array}$$

Here, the final node embedding *h*_*i*_ can be interpreted as the optimally weighted combination of relation-specific node embeddings, while each relation-specific node embedding is an optimally weighted combination of the node embeddings that share the same relation.

### 3.4. Recipe Recommendation

Above we discuss how to propagate and learn node embedding *h*_*i*_ in layer *l*+1. However, how to take advantage of these informational node embeddings and make recipe recommendations remains a challenge. In this section, we introduce how we leverage these embeddings to make proper recommendations. Specifically, suppose we propagate and update the node embeddings through *L* layers of GNN with both node-level and relation-level attentions encoded, where *L* is a hyperparameter, we obtain *L* representations generated by each layer for each node. For example, given a node *v*_*i*_ with type ϕ_*i*_, the learned node embedding $\hat{h_{i}}$ can be denoted as $\left\{h_{\phi_{i}}^{1},h_{\phi_{i}}^{2},\ldots ,h_{\phi_{i}}^{L}\right\}$, where ϕ_*i*_∈{*ins, ing, user*}. The process can be formulated as follows:


(11) $$\begin{array}{l} \hat{h_{i}}=\{\begin{array}{ll} \left\{h_{ins}^{1},h_{ins}^{2},...,h_{ins}^{L}\right\}, & \text{if }\phi_{i}=\text{recipe}\\ \left\{h_{ing}^{1},h_{ing}^{2},...,h_{ing}^{L}\right\}, & \text{if }\phi_{i}=\text{ingredient}\\ \left\{h_{user}^{1},h_{user}^{2},...,h_{user}^{L}\right\}, & \text{if }\phi_{i}=\text{user} \end{array} \end{array}$$

Since the representations generated by different layers underline the combination of messages passed over different orders of connections, they represent the node information from different perspectives. As such, they have different contributions in reflecting the node information. Therefore, we concatenate them to develop the final embedding for each node. The concatenation process can be shown as follows:


(12) $$\begin{array}{l} \hat{h_{i}}=\{\begin{array}{ll} \overset{L}{\underset{l=1}{∥}}h_{ins}^{l}, & \text{if }\phi_{i}=\text{recipe}\\ \overset{L}{\underset{l=1}{∥}}h_{ing}^{l}, & \text{if }\phi_{i}=\text{ingredient}\\ \overset{L}{\underset{l=1}{∥}}h_{user}^{l}, & \text{if }\phi_{i}=\text{user} \end{array} \end{array}$$

where *l* indicates the layer and ∥ is the concatenation operation. Accordingly, we not only enrich the last layer of node embedding with the embedding from the former propagation layers but also enable the power to supervise the range of propagation by controlling the parameter *L*. Here, we only apply concatenation to combine these different layers of embeddings out of simplicity and effectiveness (Xu et al., 2018). Still, other operations can also be leveraged such as max pooling, LSTM (Hochreiter and Schmidhuber, 1997), or weighted average. These aggregators suggest different assumptions in aggregating the embeddings. We left this to explore in future work.

Finally, we leverage a score predictor to make recommendations based on the learned user embeddings and recipe embeddings. In particular, given a user *u* with embedding *h*_*u*_ and a recipe with embedding *h*_*r*_, the score predictor can take them as the input and generate a score to indicate if the model should recommend this recipe to the user. The score is ranged between 0 and 1. When the score is closer to 1, it means the recipe should be recommended and vice versa. The recommendation process is demonstrated as follows:


(13) $$\begin{array}{l} s_{u,r}=sp\left(h_{u},h_{r}\right), \end{array}$$

where *sp* is the score predictor and *s*_*u, r*_ is the recommendation score of recipe *r* to user *u*. We further compare multiple functions for *sp* including inner product, cosine similarity, and multi-layer perceptron. The one that renders the highest performance is selected for our model (i.e., inner product). Further details are illustrated in section 4.7.3.

To learn the model parameters, we employ a ranking-based objective function to train the model. In particular, the objective involves comparing the recommendation scores between nodes connected by a *user-recipe* relation against the scores between an arbitrary pair of user and recipe nodes. For example, given an edge connecting a user node *u* and a recipe node *r*, we encourage the recommendation score between *u* and *r* to be higher than the score between *u* and a randomly sampled negative recipe node *r*′. We formulate the objective *L* as follows:


(14) $$\begin{array}{l} L=\underset{u\in U,r\in N_{u}}{\sum }max\left(0,1-s_{u,r}+s_{u,r^{\prime }}\right), \end{array}$$

where *s*_*u, r*_ and $s_{u,r^{\prime }}$ are the recommendation score between user *u* and correct recipe *r* as well as incorrect recipe *r*′, respectively, *U* denotes the user set, and *N*_*u*_ indicates the recipe neighbors of user *u*.

## 4. Experiments

In this section, we conduct extensive experiments with the aim of answering the following research questions:

- **RQ1**: How does *HGAT* perform compared to various baseline methods on recipe recommendation?
- **RQ2**: How do different components, e.g., node-level attention or relation-level attention affect the model performance?
- **RQ3**: How do various hyper-parameters, e.g., number of embedding dimensions and propagation layers, impact the model performance?

### 4.1. Dataset

We extract the recipes from Reciptor (Salvador et al., 2017; Li and Zaki, 2020) and only use those recipes that have comprehensive information (i.e., with the quantity and unit indicated for each ingredient). All these recipes are collected from *Food.com*, which is one of the largest recipe-sharing platforms online. To formulate the real-life user-recipe interactions, we crawl the user ratings for each recipe from the platform and leverage this information for the recipe recommendation task. For each ingredient that appeared in the dataset, we further match them to the USDA nutritional dataset (U.S. Department of Agriculture, 2019) to get the nutritional information. Next, we construct our recipe graph by transforming the recipes, users, and ingredients into nodes with the type “*recipe,” “user,”* and “*ingredient,”* respectively. After that, we build four types of edges among these nodes to connect them. In particular, we first connect each recipe and its ingredients with an edge, denoted as recipe-ingredient relation, while the weight of each ingredient is used as the edge weight. We then connect recipe nodes by their similarity from FoodKG (Haussmann et al., 2019) and the score is used as the edge weight, as shown in Reciptor (Li and Zaki, 2020). We further connect ingredient nodes by the co-occurring probabilities using Normalized Pointwise Mutual Information (NPMI) (Bouma, 2009) from FlavorGraph (Park et al., 2021), as shown in the KitcheNette (Park et al., 2019). Moreover, we construct edges between users and recipes based on the interactions, while the ratings are treated as the edge weight. The statistics of the constructed recipe graph are provided in Table 1.

**Table 1:** The statistics of the build recipe graph.

**Component**	**Name**	**Number**	**Data Source**
Nodes	User	7,959	Food.com
	Recipe	68,794	Reciptor [Li and Zaki (2020)]
	Ingredient	8,847	Reciptor [Li and Zaki (2020)]
Edges	User-recipe	135,353	Food.com
	Recipe-recipe	647,146	FoodKG [Haussmann et al. (2019)]
	Recipe-ingredient	463,485	Reciptor [Li and Zaki (2020)]
	Ingredient-ingredient	146,188	FlavorGraph [Park et al. (2021)]

### 4.2. Experimental Setup

We employ the leave-one-out method to evaluate the model performance, which is widely utilized in existing recommendation studies (He et al., 2016, 2017; Bayer et al., 2017; Jiang et al., 2019). Specifically, for each user, we leave one positive recipe out as the test data, one positive recipe out for validation, and used the remaining positive recipes for training. In the testing period, we randomly sampled 100 negative recipes for each user and evaluated the model performance using *Recall* and *Mean Reciprocal Rank* (MRR) metrics. We reported the performance under top@*K*, while *K* ranges from 1 to 10. The definitions of these two metrics are illustrated as follows:

- **Recall@K**. It shows the ratio of correct recipes being retrieved in the top@*K* recommendation list, which is computed by:

(15) $$\begin{array}{l} Recall@K=\frac{1}{\left|U_{test}\right|}\underset{u\in U_{test}}{\sum }\frac{\left|\mathrm{RE}\left(u\right)\cap \mathrm{GT}\left(u\right)\right|}{\left|\mathrm{GT}\left(u\right)\right|} \end{array}$$

where *U*_*test*_ is the set of users in test data for evaluation, RE(u) indicates the top@*K* recommendation list for user *u*, and GT(u) denotes the ground truth recipe set for user *u*.

- **MRR@K**. It measures the ranking quality of the recommendation list, whis is defined as:

(16) $$\begin{array}{l} MRR@K=\frac{1}{\left|U_{test}\right|}\underset{u\in U_{test}}{\sum }\frac{1}{\left|\mathrm{GTK}\left(u\right)\right|}\underset{v\in \mathrm{GTK}\left(u\right)}{\sum }\frac{1}{\hat{r}\left(v\right)} \end{array}$$

where GTK(u) denotes the ground truth recipes that appear in the top@*K* recommendation list for user *u*, and $\hat{r}\left(v\right)$ represents the ranking position of the recipe in the recommendation list.

### 4.3. Baseline Methods

We compare *HGAT* with seven baseline methods, including classic recommendation approaches, recipe representation learning methods, and graph embedding models.

- **BPR** (Rendle et al., 2009): A competitive pairwise matrix factorization model for recommendation, which is also one of the state-of-the-art algorithms used widely in recipe recommendation task (Trattner and Elsweiler, 2019).
- **IngreNet** (Teng et al., 2012): A recipe recommendation approach by replacing the popular ingredient list with the co-occurrence count extracted from the constructed ingredient network. A GCN layer is applied on the user-recipe graph to learn the embeddings jointly for a fair comparison.
- **NeuMF** (He et al., 2017): One of the state-of-the-art neural collaborative filtering models that use neural networks on user and item embeddings to capture their nonlinear feature interactions.
- **matapath2vec** (Dong et al., 2017): A heterogeneous graph embedding method based on random walk guided by meta-path. Here, we use meta-path *user-recipe-ingredient-recipe-user*.
- **GraphSAGE** (Hamilton et al., 2017): A graph neural network model that learns embeddings by aggregating and sampling the features of local neighbors.
- **GAT** (Velickovic et al., 2018): A graph attention network model that leverages the attention mechanism to aggregate neighbor information on the homogeneous graphs.
- **Reciptor** (Li and Zaki, 2020): One of the state-of-the-art recipe embedding models based on the set transformer and optimized by an instructions-ingredients similarity loss and a knowledge graph based triplet loss. A GCN layer is applied to each relation to jointly train user and recipe embedding for a fair comparison.

### 4.4. Implementation Details

For the proposed model *HGAT*, we set the learning rate to 0.005, the number of node-level attention heads to 4, the hidden size to 128, the input dimension of skip-instruction embeddings to 1,024, the input dimension of ingredient embeddings to 46, batch size to 1,024, and the training epochs to 100. We optimize the model with Adam (Kingma and Ba, 2014) and decay the learning rate exponentially by γ = 0.95 every epoch. For random walk based graph representation learning algorithms including DeepWalk and metapath2vec, we set the window size to 5, walk length to 30, the number of walks rooted at each node to 5, and the number of negative samples to 5. For homogeneous graph representation learning approaches including DeepWalk, GAT, and GraphSage, we ignore the heterogeneity of nodes and perform the algorithm on the whole graph. For a fair comparison, we set the embedding dimension to 128 for all above models except for Reciptor as we follow the original setup and use 600 as the embedding dimension.

### 4.5. Performance Comparison (RQ1)

We use the Recall and MRR as the evaluation metrics. The performances of all models are reported in Table 2. The best results are highlighted in bold. According to the table, we can find that our model *HGAT* outperforms all the baselines in all cases. Specifically, traditional collaborative filtering recommendation approaches such as BPR and NeuMF perform poorly because they neither consider the hidden relational information nor the ingredients associated with each recipe. Recipe recommendation model IngreNet obtains decent performance after incorporating the GCN to leverage the relational information. Reciptor fails to perform well because the model only learns representations for recipes while overlooking the user information. Even applying a GCN layer to learn the user embeddings jointly cannot fully encode the information. However, homogeneous graph representation learning approaches (i.e., GraphSage and GAT) achieve satisfactory results for Recall but perform poorly for MRR. This is because the node type information is important in modeling the graph structure. Ignoring this information prevents the model from learning comprehensive node embeddings and failing to rank the correct recipe higher within the returned recommendation list. On the contrary, metapath2vec, as a heterogeneous graph representation learning algorithm, performs well for both Recall and MRR. Finally, the proposed model, *HGAT*, achieves the best performance compared to all the baseline methods by incorporating recipe content, high-order interactions, relational information, and leveraging attention mechanisms to encode different types of nodes and relations. In general, compared to the best baseline, *HGAT* improves the recall score by +5.42%, +5.32%, +6.40%, +6.41%, +7.01%, +7.18%, +7.88%, +8.42%, +9.06%, and +9.53% on *k* ranges from 1 to 10, respectively. When it comes to MRR score, *HGAT* improves the score by +5.42%, +5.38%, +5.73%, +5.91%, +6.18%, +6.26%, +6.42%, +6.49%, +6.58%, and +6.67% on *k* ranges from 1 to 10, respectively. This demonstrates that *HGAT* can obtain better recipe recommendations compared to other models.

**Table 2:** Performances of different models for Top@*K* recipe recommendations.

**Metric**	**Model**	**K**									
		**1**	**2**	**3**	**4**	**5**	**6**	**7**	**8**	**9**	**10**
Recall@K	BPR	2.78	4.06	5.21	6.45	7.53	8.36	9.18	10.20	11.01	12.15
	IngreNet	9.49	13.56	16.22	18.38	19.89	21.72	23.04	24.60	26.05	27.24
	NeuMF	2.46	4.64	6.37	8.19	10.06	11.57	13.10	14.61	15.96	17.29
	metapath2vec	6.96	11.67	15.76	19.06	21.31	23.53	25.23	26.82	28.43	29.97
	GraphSage	2.55	5.40	8.23	10.91	13.70	15.99	18.62	21.08	23.38	25.56
	GAT	2.89	5.91	8.96	11.82	14.55	17.49	20.02	22.58	25.12	27.33
	Reciptor	8.63	12.33	14.1	15.22	16.04	16.85	17.77	18.61	19.2	19.91
	HGAT	**14.91**	**18.88**	**22.62**	**25.47**	**28.32**	**30.71**	**33.11**	**35.24**	**37.49**	**39.50**
MRR@K	BPR	2.78	3.42	3.80	4.11	4.33	4.46	4.58	4.71	4.80	4.91
	IngreNet	9.49	11.52	12.41	12.95	13.25	13.56	13.75	13.94	14.10	14.22
	NeuMF	2.46	3.55	4.13	4.58	4.96	5.21	5.43	5.62	5.77	5.90
	metapath2vec	6.96	9.32	10.68	11.50	11.95	12.32	12.57	12.77	12.94	13.10
	GraphSage	2.55	3.98	4.92	5.59	6.15	6.53	6.90	7.21	7.47	7.68
	GAT	2.89	4.40	5.42	6.13	6.68	7.17	7.53	7.85	8.13	8.35
	Reciptor	8.63	10.48	11.07	11.35	11.51	11.65	11.78	11.88	11.95	12.02
	HGAT	**14.91**	**16.90**	**18.14**	**18.86**	**19.43**	**19.82**	**20.17**	**20.43**	**20.68**	**20.89**

### 4.6. Ablation Studies (RQ2)

*HGAT* is a joint learning framework composed of several neural network modules. How do different components impact the model performance? To answer this question, we conduct ablation studies to evaluate the performances of several model variants including:

- *HGAT*_*mean*_: a model variant that uses neither node-level attention nor relation-level attention. Instead, it uses a mean operator to combine the neighbor node features, and relation features.
- *HGAT*_*pool*_: a model variant that uses neither node-level attention nor relation-level attention. Instead, it uses a pooling operator to combine the neighbor node features and a mean operator to combine the relation features.
- *HGAT*_*nAtt*_: a model variant that uses node-level attention to fuse neighbor node features, and mean operator to combine the relation features.
- *HGAT*: the proposed model that leverages both node-level attention and relation-level attention.

The results are reported in Table 3. From this table:

- *HGAT*_*pool*_ has better performance than *HGAT*_*mean*_, indicating the way how to aggregate neighbor nodes information is important, and simply using the mean operator to combine the neighbor node messages could lose some information.
- *HGAT*_*nAtt*_ performs better than *HGAT*_*pool*_ and *HGAT*_*mean*_, demonstrating the effectiveness of node-level attention and illustrating using attention mechanism advances using mean or pooling operator in aggregating the neighbor nodes information.
- The proposed *HGAT* outperforms all the model variants including *HGAT*_*nAtt*_, showing that the incorporation of relation-level attention could further improve the performance. This demonstrates the effectiveness of relation-level attention.

**Table 3:** Comparison of different model variants on top@*K* recommendations.

**Metric**	**Model**	**K**									
		**1**	**2**	**3**	**4**	**5**	**6**	**7**	**8**	**9**	**10**
Recall@K	*HGAT*_*mean*_	12.50	17.33	20.76	23.46	25.79	28.03	30.41	32.49	34.33	36.07
	*HGAT*_*pool*_	13.67	18.26	21.52	24.59	27.06	29.26	31.24	33.50	35.34	37.24
	*HGAT*_*nAtt*_	14.63	18.87	22.02	25.38	28.04	30.29	32.52	34.92	36.90	38.84
	*HGAT*	**14.91**	**18.88**	**22.62**	**25.47**	**28.32**	**30.71**	**33.11**	**35.24**	**37.49**	**39.50**
MRR@K	*HGAT*_*mean*_	12.50	14.91	16.06	16.73	17.20	17.57	17.91	18.17	18.38	18.55
	*HGAT*_*pool*_	13.67	15.96	17.05	17.82	18.31	18.68	18.96	19.24	19.45	19.64
	*HGAT*_*nAtt*_	14.63	16.75	17.80	18.42	18.93	19.30	19.62	19.92	20.14	20.34
	*HGAT*	**14.91**	**16.90**	**18.14**	**18.86**	**19.43**	**19.82**	**20.17**	2**0.43**	**20.68**	**20.89**

### 4.7. Parameter Sensitivity (RQ3)

To estimate the proposed models' sensitivity to hyper-parameters, we conducted many contrast experiments to measure the performance of *HGAT* under different hyper-parameter settings. We start by exploring the influence of embedding dimensions, as it usually plays a pivotal role in data modeling. We then analyze the impact of propagation layer numbers to show the importance of modeling relational information. Moreover, we study how different score predictors affect recommendation performance.

#### 4.7.1. Impact of Different Embedding Dimensions

**Figure 4:**
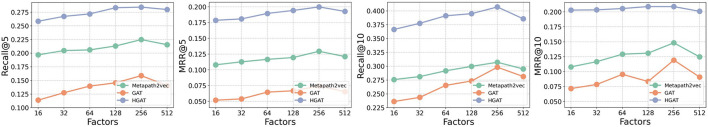
Performance of Top@10 recipe recommendations *w.r.t*. the number of embedding dimensions.

We report the performance of Recall@5, MRR@5, Recall@10, and MRR@10 with respect to the number of embedding dimensions in Figure 4. Specifically, we search the number of embedding dimensions within {16, 32, 64, 128, 256, 512} and evaluate the performance of the proposed *HGAT* and two best baselines (i.e., metapath2vec and GAT). From the figure:

- Increasing the number of embedding dimensions improves the model performance. Clearly, all models achieve the highest score on the 2 metrics when using the 256 dimensions. This is because more dimensions could have more capacity to represent the node content.
- Further increasing the number of embedding dimensions to 512 leads to overfitting. This might be projecting the representation into a higher-dimensional space introduced noise.
- When varying the number of embedding dimensions, *HGAT* is consistently superior to other methods on different setups. This demonstrates the capability of *HGAT* compared to other approaches.

#### 4.7.2. Impact of Different Propagation Layers

We vary the model depth and test performance to investigate whether the proposed *HGAT* can benefit from multiple embedding propagation layers. Specifically, we search the layer numbers in {1, 2, 3, 4}. Experimental results are reported in Table 4, wherein *HGAT*-2 indicates the model with 2 embedding propagation layers and similar notations for others. By analyzing the Table 4, we have the following observations:

- Equipping *HGAT* with more propagation layers substantially enhances the recommendation performance. Clearly, *HGAT*-2 and *HGAT*-3 achieve consistent improvement over *HGAT*-1 in all cases, given *HGAT*-1 only considers the first-order neighbor nodes. We attribute this improvement to the effective modeling of relational information: relational information are carried by second-order and third-order connectivities, and relational information can be modeled by encoding these interactions.
- When further stacking propagation layer on the top of *HGAT*-3, we find that *HGAT*-4 leads to overfitting on the dataset. This is because applying a too deep architecture might introduce noises into modeling. Similar results to *HGAT*-3 verifies that conducting three propagation layers is sufficient to capture the relational information.
- By comparing the results in Table 4 to Table 2 (which report the *HGAT*-2 performance), we can find *HGAT* is consistently superior to other methods. This again verifies the effectiveness of *HGAT*, empirically showing that explicit modeling of high-order interactions and relational information can greatly facilitate the modeling and further improve the performance in recommendation tasks.

**Table 4:** Performance of *HGAT* on Top@*K* recommendations *w.r.t*. propagation layers.

**Metric**	**Layers**	**K**									
		**1**	**2**	**3**	**4**	**5**	**6**	**7**	**8**	**9**	**10**
Recall@K	*HGAT*-1	14.57	17.62	19.15	20.47	21.70	22.92	24.24	25.54	26.95	28.13
	*HGAT*-2	14.91	18.88	22.62	25.47	28.32	30.71	33.11	35.24	37.49	39.50
	*HGAT*-3	**15.19**	**19.71**	**23.47**	26.45	29.17	**31.90**	**34.55**	**36.86**	**39.26**	**41.3**
	*HGAT*-4	14.73	19.50	23.28	**26.80**	**29.45**	31.71	34.33	36.54	38.62	41.09
MRR@K	*HGAT*-1	14.57	16.09	16.61	16.94	17.18	17.39	17.57	17.74	17.89	18.01
	*HGAT*-2	14.91	16.90	18.14	18.86	19.43	19.82	20.17	20.43	20.68	20.89
	*HGAT*-3	**15.19**	**17.45**	**18.70**	**19.45**	**19.99**	**20.45**	**20.83**	**21.12**	**21.38**	**21.59**
	*HGAT*-4	14.73	17.11	18.37	19.25	19.78	20.16	20.53	20.81	21.04	21.29

#### 4.7.3. Impact of Different Score Predictors

To show the effectiveness of the used score predictor in our model, we testify the effect of different score predictors and report the performance in Table 5. In particular, we use different similarity functions, namely, cosine similarity, multi-layer perceptron (MLP), and inner product. We evaluate the performance of different functions on Top@*K* recommendations where *K* ranges from 1 to 10. From the table:

- Inner product is the best function to calculate the similarity score and cosine is the worst. This might be because the cosine similarity only cares about the angle difference between 2 vectors, which fails to capture the complexity of the learned embeddings, while the inner product considers both the angle and the magnitude.
- MLP achieves similar performance on inner product under Recall but still performs worse when using MRR to evaluate, which is consistent with the findings in paper (Rendle et al., 2020). This further shows the compatibility of the inner product.

**Table 5:** Performance of *HGAT* on Top@*K* recipe recommendations *w.r.t*. the score predictors.

**Metric**	**Score Predictor**	**K**									
		**1**	**2**	**3**	**4**	**5**	**6**	**7**	**8**	**9**	**10**
Recall@K	Consine	14.89	17.67	19.96	22.01	24.04	26.08	28.19	30.10	32.23	34.15
	MLP	14.21	18.53	22.16	24.89	27.48	29.89	32.16	34.41	36.69	38.71
	Inner Product	**14.91**	**18.88**	**22.62**	**25.47**	**28.32**	**30.71**	**33.11**	**35.24**	**37.49**	**39.50**
MRR@K	Cosine	14.89	16.33	17.09	17.61	18.01	18.35	18.65	18.89	19.13	19.32
	MLP	14.21	16.37	17.58	18.26	18.78	19.18	19.51	19.79	20.04	20.24
	Inner Product	**14.91**	**16.90**	**18.14**	**18.86**	**19.43**	**19.82**	**20.17**	**20.43**	**20.68**	**20.89**

### 4.8. Case Study: Embedding Visualization

**Figure 5:**
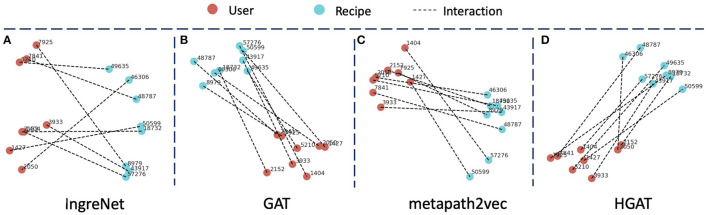
Visualization of the user and recipe embeddings generated by different models. Red nodes represent the user while blue nodes represent the recipe. An edge connecting a user and a recipe indicates the interaction between them.

For a more intuitive understanding and comparison, we randomly select 8 user-recipe interaction pairs and generate the visualization of their embeddings using t-SNE (van der Maaten and Hinton, 2008). As shown in Figure 5, we can find that IngreNet does not perform well. The model can roughly separate the users and recipes into the left and right parts, but there are gaps within each cluster. Also, the lines between clusters are disordered. Ideally, there should develop a clear mapping between each user-recipe pair. In other words, if we connect for each pair, the connecting lines should be parallel to each other [similar to the “king-man=queen-woman” relationship (Mikolov et al., 2013)]. GAT can successfully form the users and recipes into two condense clusters, but fail to construct the parallel lines between them. Metapath2vec builds two clusters for users and recipes and establish parallel lines between clusters in some sense. Examples are the “1404–57276” and “1427–50599” user-recipe pairs. However, the formed clusters are not condensed, and only a few of the lines are parallel to each other. Finally, our model *HGAT* can easily form the users and recipes into 2 condensed clusters and obtain parallel lines for almost all user-recipe pairs. This further demonstrates the superiority of our model.

## 5. Conclusion

In this paper, we propose to leverage the relational information into recipe recommendation. To achieve this, we design *HGAT*, a novel hierarchical graph attention network for solving the problem. *HGAT* is able to capture user history behaviors, recipe content, and relational information through several neural network modules. We further introduce a score predictor and a ranking-based objective function to optimize the model. Extensive experiments demonstrate that *HGAT* outperforms numerous baseline approaches. In the future, we plan to incorporate more information and improve *HGAT*. We observe that there are still plenty of useful information components that we can use such as user reviews and recipe health factors. One promising direction is to investigate how to make recipe recommendation that fit user preferences and health concerns.

## Data Availability Statement

The raw data supporting the conclusions of this article will be made available by the authors, without undue reservation.

## Author Contributions

YT, CZ, RM, and NC contributed to the overall design of the study. YT conducted the experiments. CZ performed the interpretation of results. YT wrote the first draft of the manuscript. All authors contributed to manuscript revision and approved the submitted version.

## Funding

This work was supported by the Agriculture and Food Research Initiative grant no. 2021-67022-33447/project accession no.1024822 from the USDA National Institute of Food and Agriculture.

## Conflict of Interest

The authors declare that the research was conducted in the absence of any commercial or financial relationships that could be construed as a potential conflict of interest.

## Publisher's Note

All claims expressed in this article are solely those of the authors and do not necessarily represent those of their affiliated organizations, or those of the publisher, the editors and the reviewers. Any product that may be evaluated in this article, or claim that may be made by its manufacturer, is not guaranteed or endorsed by the publisher.

## Footnotes

^1^ https://www.food.com

^2^ https://github.com/guoguibing/librec
